# Estimates for local and movement-based transmission of bovine tuberculosis in British cattle

**DOI:** 10.1098/rspb.2007.1601

**Published:** 2008-02-12

**Authors:** Darren M Green, Istvan Z Kiss, Andrew P Mitchell, Rowland R Kao

**Affiliations:** 1 Institute of Aquaculture, University of StirlingStirling, Stirlingshire FK9 4LA, UK; 2 Department of Mathematics, University of SussexFalmer, Brighton BN1 9QH, UK; 3 Veterinary Laboratories AgencyWeybridge, New Haw, Addlestone, Surrey KT15 3NB, UK; 4 Institute of Comparative Medicine, Faculty of Veterinary Medicine, University of GlasgowGlasgow G61 1QH, UK

**Keywords:** epidemic, *Mycobacterium bovis*, breakdown

## Abstract

Both badgers and livestock movements have been implicated in contributing to the ongoing epidemic of bovine tuberculosis (BTB) in British cattle. However, the relative contributions of these and other causes are not well quantified. We used cattle movement data to construct an individual (premises)-based model of BTB spread within Great Britain, accounting for spread due to recorded cattle movements and other causes. Outbreak data for 2004 were best explained by a model attributing 16% of herd infections directly to cattle movements, and a further 9% unexplained, potentially including spread from unrecorded movements. The best-fit model assumed low levels of cattle-to-cattle transmission. The remaining 75% of infection was attributed to local effects within specific high-risk areas. Annual and biennial testing is mandatory for herds deemed at high risk of infection, as is pre-movement testing from such herds. The herds identified as high risk in 2004 by our model are in broad agreement with those officially designated as such at that time. However, border areas at the edges of high-risk regions are different, suggesting possible areas that should be targeted to prevent further geographical spread of disease. With these areas expanding rapidly over the last decade, their close surveillance is important to both identify infected herds quickly, and limit their further growth.

## 1. Introduction

Bovine tuberculosis (BTB) control in Great Britain (GB) cost over £90 million in 2005 including £35 million in compensation to cattle farmers (http://www.defra.gov.uk/animalh/tb/stats/expenditure.htm). Disease spread at the national level is due to both cattle movements and other factors. Particularly unwelcome is transmission from infected badgers, generally resulting in ‘high-risk’ areas where cattle are at greater risk of becoming infected (Wint *et al*. 2002; Woodroffe *et al*. 2006). While recent changes in government policy have introduced tuberculin testing for cattle prior to movement from herds deemed at high risk (http://www.defra.gov.uk/animalh/tb/pdf/pre-movementtest.pdf), the relative importance of cattle movements remains to be assessed.

Transmission routes of *Mycobacterium bovis* to cattle are multiple, but poorly quantified (Menzies & Neill 2000; Goodchild & Clifton-Hadley 2001; Neill *et al*. 2001). Movements of infected cattle have been shown to pose a clear transmission risk (Goodchild & Clifton-Hadley 2001; Gopal *et al*. 2006), and cattle movements are a significant predictor of the distribution of BTB (Gilbert *et al*. 2005). In some high-risk areas of GB, *M. bovis* is also widespread in badgers (Gallagher & Clifton-Hadley 2000; de la Rua-Domenech *et al*. 2006), and they are implicated as a wildlife reservoir, as is the case for wildlife carrying *M. bovis* elsewhere (Morris *et al*. 1994; Delahay *et al*. 2002; Ramsey *et al*. 2002; Griffin *et al*. 2005; O'Brien *et al*. 2006). However, badger culling trials (Donnelly *et al*. 2006; Woodroffe *et al*. 2006) have produced some complex results, and thus quantification of the relative impact of movement-based and risk-area-related infection is critical for determining future control policies.

Cattle herds in GB are tested every 1, 2, 3 or 4 years for BTB depending on criteria laid down by EU directive 64/432/EEC and the perceived local risk of herd infections, generally referred to as ‘breakdowns’ (see electronic supplementary material, figure A1). As cattle movements are a transmission risk (Goodchild & Clifton-Hadley 2001; Gilbert *et al*. 2005; Gopal *et al*. 2006), pre-movement testing has been introduced for cattle aged over six weeks (March 2006; initially for over 15 months until March 2007) moving out of parishes with 1- and 2-year testing intervals, and from a few other specified high-risk herds. The cost of this policy (up to £6 million per annum) prompts two critical questions: what proportion of transmission can be attributed to movements, and can current protocols for identifying pre-movement testing areas and designation of high-risk herds be improved? We address these questions via a model that attributes breakdowns to three causes. First, transmission is modelled through recorded movements. Second, infection occurs within high-risk areas including due to wildlife reservoirs and direct cattle-to-cattle spread between contiguous herds. Third, additional countrywide ‘background’ risk of breakdown through other causes is modelled, potentially accounting for unrecorded movements and contact with infected cattle occurring outside of high-risk areas. We consider two models for the high-risk areas, first based upon annual and biennial testing areas (i.e. parish-based or parochial high-risk areas), and second assuming a fitted radius of high-risk surrounding known BTB cases identified over 1 year (2003), to determine whether their spatial locations are good markers for the true extent of areas at high risk of BTB spread in the following year.

## 2. Material and methods

We use cattle tracing system (CTS) data provided by RADAR (http://www.defra.gov.uk/animalh/diseases/vetsurveillance/radar/), details of BTB breakdowns reported to DEFRA's animal health database, VetNet, and the June Agricultural Survey for 2003 (http://www.defra.gov.uk/esg/work_htm/publications/cs/farmstats_web/default.htm) as detailed in the electronic supplementary material. The movement data were consolidated into batches of cattle moved between pairs of georeferenced premises on given dates between 2002 and 2005 (see electronic supplementary material for georeferencing details). In brief, the model considered 130 755 locations, with 3 624 643 processed batch movements of cattle during this period, of mean size 3. Over the same time period, there were 7425 confirmed breakdowns recorded by VetNet over 6139 different premises.

The model is based upon a previously developed framework for modelling livestock disease transmission through movements and other mechanisms (Green *et al*. 2006; Kao *et al*. 2006). It operates at the level of the premises and is spatially explicit, using 1-day time steps. Breakdowns from 2003 in 1- and 2-year testing areas were used to set the initial state of the model (index cases). Premises *i* at time *t* is infected with probability *p*_*i*,*t*_. This probability is amended when the premises is exposed to infection through one of the three modelled routes (see electronic supplementary material for full details).

### (a) Cattle movements

Cattle movements are a known BTB risk factor (Gilbert *et al*. 2005). Movements from infected premises are infectious with probability *μ* per animal moved. We consider two possibilities: in the ‘high within-herd’ transmission model, all premises exposed to cattle that have been resident in high-risk areas are themselves a risk, so *μ* applies to all cattle moving from exposed herds. In the ‘low within-herd’ transmission model, *μ* only applies to those cattle that have previously passed through high-risk areas.

### (b) High-risk areas

We assume areas with endemic BTB to be at higher risk of infection. Little is known of within-premises dynamics to distinguish among premises types; therefore, we assume all premises within specified high-risk areas as subject to infection with constant daily probability *γ*/*n*, where *n* is the number of premises in high-risk areas. Two types of high-risk areas are defined: all premises in parishes with 1- or 2-year testing intervals (‘parochial’ high-risk areas); or all premises within a radius *r* of an index case.

### (c) Background rate

All premises are subject to infection with a constant daily probability *β* to account for cases not explained by the other two factors.

Maximum-likelihood parameter estimates for *μ*, *β*, *γ*, *r* were obtained as described in the electronic supplementary material, with confidence limits provided by a Markov chain Monte Carlo algorithm.

## 3. Results

The proportions of infections due to movements, background rate spread, and presence in high-risk areas, and with corresponding transmission rates *μ*, *β*, *γ* and radius *r* are shown in table 1 and figure 1 (and figure A1 in the electronic supplementary material) for different modelling assumptions, using the year 2003 for model seeding, and the year 2004 for evaluation of the model likelihood function (given in the electronic supplementary material). Parameter *w*, the assumed possible window of infection prior to discovery was set at 1 year, requiring a model start date at the beginning of 2002. Models were fit using a maximum-likelihood method and compared via the Akaike information criterion (AIC; Akaike 1974). Where models were nested (high within-herd versus low within-herd, background versus no background), statistical significance was confirmed using likelihood ratio tests. The best-fit model assumed high-risk areas based on radii surrounding index cases, and low within-herd transmission, where only cattle that have stayed on premises in high-risk areas are assumed potentially infectious. Movement accounted for 16% of infections, with background infection 9%, and the remaining 75%, due to high-risk area transmission. The 95% CIs are narrow with confidence in the proportion of movement-related infections notably strong (figure 1). Model results were insensitive to the model start date and the infection window *w* (duration of infectiousness before reported breakdown) within the range of 70–365 days (figure A1 in the electronic supplementary material) due to repeated sampling from the high-risk areas (figure A2 in the electronic supplementary material).

VetNet data include both confirmed (via culturing of *M. bovis* or appearance of lesions typical of BTB) and unconfirmed cases. Including the unconfirmed cases in the analysis, the results (not shown) differed in an increased contribution of background spread of approximately 15%. The increase in ‘unexplainable’ breakdowns suggests that the majority of unconfirmed breakdowns are a low risk for onward transmission, either because they are inherently less infectious or more likely because they represent false-positive tests.

As a further test of model robustness, model fits were obtained with all time frames moved forwards 1 year, such that 2004 breakdowns were used to seed the model and the model output fitted against 2005 breakdown data, using the appropriate movement data. Results for the low within-herd transmission model were similar to those obtained for the 2003–2004 model fit, as shown in the electronic supplementary material (table A1 in the electronic supplementary material).

The model assumes that all premises included in the model are equally susceptible to infection given equal levels of exposure. As there are known risk factors (Gilbert *et al*. 2005), it is probable that breakdown farms are in some way more susceptible. To investigate this, breakdown herds were assumed inherently more susceptible than all other herds and the model refitted (figures A3 and A4 in the electronic supplementary material). This improved model fit, but did not substantially change the proportions of infection through the three routes (see electronic supplementary material), as all the risk factors increase proportionally.

The current control policy in GB requires annual or biennial herd testing and pre-movement testing in specified, high-risk parishes. Our results suggest that attribution of risk by areas centred on breakdown herds in 2003 would have identified 0.5% more of BTB-infected herds in 2004, reducing the infected herds not in these areas by 20% (table 2). This model has a lower AIC compared with models with parochial risk. However, since 1998, frequent testing areas have expanded dramatically in England and Wales (figure A5 in the electronic supplementary material), and the benefits of replacing the simply implemented parochial testing scheme with a more complicated approach would be small, if this is the measure used.

To test whether radial test areas perform better than parochial areas simply because they encompass more herds (figure 2), we replaced index cases with other premises selected randomly from the same parish, and compared radius-based high-risk areas centred on the breakdown herds with those centred on the randomized index cases. Similar parameters were thereby obtained, though model selection based on the AIC showed the models based on randomized cases to be substantially worse (table 1).

The cumulative effect of transmission mechanisms is nonlinear, so to test the possible effect of control strategies, we selectively reduce each parameter to zero, rerun the model, and determine the effect on breakdown rate. Complete inactivation of all movement and background spread caused the maximal reduction of 24% simulated breakdowns in 2004. Selectively inactivating movement-related transmission alone in the best-fit model (low within-herd transmission) reduced the number of simulated herd breakdowns by 14.6% where movements from 1- and 2-year testing areas are not considered infectious (an estimated 323 breakdowns, corresponding to 93% of movement-related infections). This assumes no exemptions, and perfect testing of all cattle of all ages, but does not include cases accounted for under background rate spread. Using radius-based high-risk areas caused a greater reduction of 15.4% of breakdowns (98% of movement-related infections).

Owing to the different time frames, and because our model includes all movement-based spread, formal comparisons with pre-movement testing data are not possible, however, in the first seven months of pre-movement testing, 59 confirmed breakdowns were identified by the scheme. In addition, current policy requires pre-movement testing from certain premises not in 1- or 2-year testing parishes, which will reduce the risk of onward transmission further. We also reduce the rate parameter for high-risk area spread to zero. In the radial high-risk areas, this reduces simulated nationwide 2004 incidence by 81%, including a 37% reduction in further spread onwards through movements.

## 4. Discussion

The three modelled components encompass various routes of transmission. The high-risk area process could account for both direct farm-to-farm spread and spread from wildlife reservoirs. The background rate will include unrecorded movements, infectious movements that predate those used by the model, and other long-distance transmission mechanisms such as fomites. It will also account for some overspill from high-risk areas. It does, however, make the implicit assumption that these processes occur at equal rate across the country. Little external data are available to quantify the proportions of these three ‘routes’ of transmission. In one earlier study (Wilesmith 1983; MAFF 1991), 89% could not be attributed to cattle movements. However, management practices will have changed considerably since this time. In contrast, badger removal in cull trials led to a decrease in confirmed incidence of only 19% compared with control areas (Donnelly *et al*. 2006), but was for several reasons an incomplete cull: it can only be seen as an estimated lower bound for breakdowns from a badger source.

Aside from the established links between BTB infection in badgers and cattle (Woodroffe *et al*. 2006), unrecorded movements of cattle and earlier movements (e.g. prior to 2002), contiguous grazing areas with infected cattle, and transmission due to human activities would all be expected to contribute to transmission in high-risk areas. However, they would also contribute similarly in areas of low risk provided infected herds in both high- and low-risk areas are otherwise similar. Where risk factors (e.g. grazing practices) are clustered, they will be represented mainly as part of the high-risk area spread. Under our assumption that the background process *β* is similar in both high- and low-risk areas, transmission due to *β* only accounts for 3% (54) of breakdowns in 2004 in high-risk areas. Outside of high-risk areas, background transmission accounts for 50% of breakdowns. This is consistent with a detailed investigation of a small cluster of breakdowns in northeast England (Gopal *et al*. 2006) where 16 out of 31 breakdowns contained at least one confirmed case traced from a breakdown herd with a matching molecular type of *M. bovis*.

Our model is fitted only to the observed epidemic and does not consider cattle harbouring *M. bovis* but missed either through never having being tested or owing to test insensitivity (de la Rua-Domenech *et al*. 2006). However, in the absence of biases in these missing data, our estimate of the relatively low importance of cattle movements should be robust. It is also consistent with prior results showing that cattle testing alone can control cattle-to-cattle spread (Kao *et al*. 1997), and that few breakdown herds in low-risk areas contain infected homebred cattle (Gopal *et al*. 2006). Our model also assumes that BTB is a single homogeneous infectious agent across GB. This is a simplification: BTB exists in GB as numerous genotypes, most with a high degree of geographical clustering (Smith *et al*. 2006). Further work is required to determine the effect of incorporating such data into the model developed above, and whether there are identifiable inhomogeneities across the genotypes.

High-risk spread is probably the result of the cattle–badger–BTB interaction, though there is potential for contributions from other clustered risk factors. Evidence that cattle-to-badger transmission is important is sparse (Woodroffe *et al*. 2006), however if true, seedings via cattle infections may contribute to the broadening of high-risk areas (figure A5 in the electronic supplementary material). Our model identifies significant areas that would not be under restriction given current protocols, suggesting that closer investigation is necessary for other areas not currently under restriction. While there appears to be little immediate benefit in terms of a reduction in herd breakdowns, other benefits of close surveillance must also be considered: close surveillance of high-risk areas will not only help to prevent spread to low-risk areas but it may also help to control the growth of the high-risk areas themselves.

## Figures and Tables

**Figure 1:**
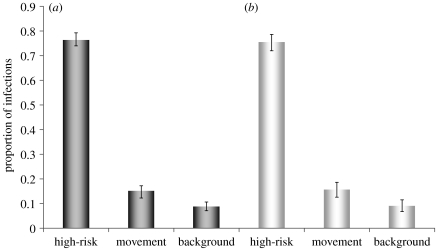
CIs. Estimates and 95% profile CIs for proportions of infections caused by movement, background and high-risk area transmission. Model likelihood was evaluated at the end of 2004 for (*a*) the high within-herd and (*b*) low within-herd transmission models.

**Figure 2:**
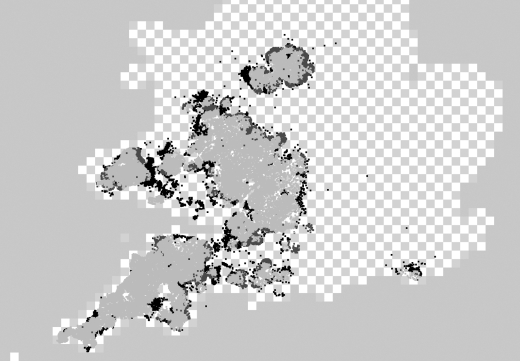
Distributions of high-risk areas as estimated for 2004 (low within-herd spread model). Premises in both radius-based and parochial high-risk areas are shown in light grey, premises only in radius-based areas in dark grey, and only in parochial-based areas in black. Elsewhere is shown with a checked background.

**Table 1:** Best-fit models. (All model runs were for *w*=365 days and a model start point of January 2002. Akaike information criterion (AIC) values presented are the mean AIC statistics for each day of 2004. B, Background rate spread fitted; P, parish-based high-risk areas fitted; R, radius-based high-risk areas fitted; M, movement transmission fitted. a, High-risk areas based on the ‘true’ index cases; b, average of fits for five different sets of randomized index cases; c, High-risk areas based on the set of randomized index cases used for each simulation.)

		parameters				
			
model		moves *μ*	background *β*	high-risk *γ*	radius *r* (m)	AIC
high within-herd	B	—	15.1×10^−6^	—	—	18 878
	BM	2.54×10^−3^	11.7×10^−6^	—	—	18 330
	PM	4.61×10^−3^	—	2.08	—	16 252
	PBM	3.38×10^−3^	1.86×10^−6^	2.01	—	15 642
	RM	2.42×10^−3^	—	1.70	10 300	15 887
	RBM	2.12×10^−3^	1.37×10^−6^	1.60	5987	15 508
low within-herd	RBM	2.49×10^−3^	1.41×10^−6^	1.59	6000	15 489
randomized index	RaBMb	2.62×10^−3^	1.26×10^−6^	1.77	6000	16 121
cases	RcBMb	2.70×10^−3^	1.31×10^−6^	1.74	5715	16 162

**Table 2:** Distributions of 2004 BTB case premises across radius- and parish-based high-risk areas (low within-herd spread model), showing cases in both, neither, or one type of area. (The proportions of all premises in or out of a given high-risk area that were BTB cases in 2004 are given in brackets.)

	parochial high-risk areas		
	
radius-based high-risk areas	inside	outside	total
inside	1497	49	1546 (1.18%)
outside	39	161	200 (0.15%)
total	1536	210	1746
	(1.17%)	(0.16%)	

## Supplementary Material

Electronic appendix Supplementary background, methods, and results
