# Supplementary material for: Estimates for local and movement-based transmission of bovine tuberculosis in British cattle
Source: Proc Biol Sci. 2008 Feb 12;275(1638):1001–5. doi: 10.1098/rspb.2007.1601 (PMC2366193; doi:10.1098/rspb.2007.1601)
Supplement: Supplementary file 1 [file rspb20071601s01.pdf]

Estimates for local and movement-based transmission of bovine tuberculosis in British Cattle — Electronic Appendix.

Darren M. Green, Istvan Z. Kiss,
Andrew P. Mitchell and Rowland R. Kao

February 15, 2008

A1. Background

Cattle herds in Great Britain (GB) are ascribed a testing interval, with herds tested every one, two, three or four years for bovine tuberculosis (BTB) depending on criteria laid down by European Union directive 64/432/EEC. The administrative unit for testing intervals is the parish (numbering approximately 12 000), such that all herds in a parish will be tested at the same frequency, unless a herd is perceived to be at a higher risk of infection, such as some dealer herds, herds contiguous with TB incident herds, or herds presenting a heightened public health risk. For these (804 as of late 2006), a high test frequency will apply.

Parish testing intervals are simple to implement, and are easily understood by farmers; however, parishes are not standardised by shape, size, or number of holdings, so their boundaries will not reflect the geographical extent of infection with any precision. Furthermore, testing intervals are reviewed annually, using recent breakdown histories to determine whether a change is required (Fig. A3). Nevertheless, the review of testing intervals in the years following the 2001 outbreak of foot-and-mouth disease through to 2004 was atypical, and since 2005 has been carried out more methodically.

A2. Source data

The source data used were from the Cattle Tracing System (CTS) of GB, provided by RADAR (Rapid Analysis and Detection of Animal-related Risks `http://www.defra.gov.uk/animalh/diseases/vetsurveillance/radar/`), and details of BTB breakdowns (cases) as reported to DEFRA's animal health database, VetNet (`http://www.defra.gov.uk/animalh/tb/stats/index.htm`). The model considered 130 755 premises identified by CTS. Of these, many had no associated geographical coordinates data, for which coordinates data were extracted from the June Agricultural Census for 2003 (`http://www.defra.gov.uk/esg/work_htm/publications/cs/farmstats_web/default.htm`). Coordinates data were then assigned to 97.8 % of premises. Coordinates for 0.7 % of premises were inferred using the mean coordinates for premises located in the same parish (as identified from county/parish/holding CPH codes), plus jitter

within 1 km^2. Another 1.3 % of premises were similarly relocated due to having coordinates data unrepresentative of their parish. A remaining 0.2 % of premises could not be located and were exempted from infection by local spread in the model. All georeferences are given as point locations. Insufficient data were available in order to robustly model below holding level (i.e. at herd level), therefore the herd portions of CPHH (county/parish/holding/herd) codes reported to VetNet were discarded.

CTS movement data are recorded as animal 'histories', a series of records of animal stays consisting of a location, a cattle ID number, and dates of arrival and departure. These stays were converted into cattle movements by matching up adjoining stays. Movements to slaughter were not considered by the model and were removed. Short-term stays at markets were also not considered as infectious, and were 'spliced' from the dataset such that movements $A \to B \to C$, where B is a market, were replaced by a single movement $A \to C$. Moves with equal dates, start-, and end-points were grouped into batches. For 2002-5, there were 3 624 643 resulting batch movements with a mean batch size of 3.

Breakdowns were identified according to date and CPH from the Vetnet data. The model is provided with those breakdown data that are recorded as 'confirmed' by Vetnet. For 2002-5, there were 7 425 such confirmed tests. Of these, 99.8 % were matchable to CPH codes present in the CTS data, across 6 139 different premises.

A3. Model construction

The model is based upon a previously developed framework for modelling livestock disease transmission through movements and other mechanisms (Green et al. 2006; Kao et al. 2006). It is individual based, at the level of the premises. Each premises i maintains a probability of infection through the simulation, P_i, updated using one-day time-steps. Each potential infection event causes infection with probability p, as calculated below. This causes an increase in P_i, conditional on the probability of i already being infected, such that

$$\Delta P = (1 - P_i)\, p$$

$$P_i \mapsto P_i + \Delta P$$

The summation of ΔP across all infection events gives the expectation of the total number of infections produced during the simulation, I, and it may be partitioned into the causes of infection listed below: total infections due to livestock movements (M), infections within high-risk areas (G) and background rate, countrywide (B). The expected prevalence at a given time is given by $\sum_i P_i$.

Livestock movement Cattle movements are a known BTB risk factor (Gilbert et al. 2005). Movements from infected premises are infectious with probability μ per animal moved, thus weighting the infectiousness of movements by the number of cattle moved c. We consider two possibilities: In the 'high within-herd' transmission model, all premises exposed to cattle that have been resident in high-risk areas are themselves a risk, so μ applies to all cattle moving from exposed herds. In the 'low within-herd' transmission model, μ only applies to those cattle that have previously passed through high-risk areas. A livestock movement from premises j to premises i is

therefore considered potentially infectious where j has a high probability of infection and where the number of animals moved is large:

$$p = \left(1 - (1 - \mu)^c\right) P_j.$$

High-risk area We assume areas with endemic BTB to be at high risk of infection. Little is known of within-premises dynamics to distinguish amongst premises types; therefore, we assume all premises within these areas are subject to infection with constant daily probability $p = \frac{\gamma}{n}$ where n is the number of premises in high-risk areas. Parameter γ is thus the mean daily rate of production of infected premises through this mechanism in a susceptible population. Two types of high-risk areas are defined: all premises in parishes with one- or two-year testing intervals ('parochial' high-risk areas); or all premises within a radius r of an index case, whose definition is described below.

Background rate Each premises is exposed to infection on a daily basis with a fixed probability β, independently of location or movement; the model considers an infection event $p = \beta$ once per day for each premises. This simulates infection due to unknown causes, not included by the other two factors.

A4. Model evaluation

Unless otherwise stated, simulations were run from the beginning of 2002 until the end of 2004. Breakdowns from 2003 in one- and two-year testing areas were used to set the initial state of the model (index cases). For each index case breakdown i, occurring at time t, premises i was considered infectious from time $t - w$, to t ($P_i = 1$). Parameter w therefore represents the unknown time elapsed between infection of a premises and its eventual detection. P is then updated by the model, as above. High-risk areas were defined as either a) all premises in one- or two-year testing areas, or b) all premises within a radius r of an index case. No higher risk was assigned to premises in overlapping radii.

Model predictions for 2004 were then tested against the data (Fig. A6). The variable Y_i represented an estimate from the breakdowns data of the premises status, assigned in a manner analogous to P. Y_i was set as $Y_i = 1$ between times $t - w$ and t where t is the time of a breakdown occurring in 2004-5, and $Y_i = 0$ otherwise. Additionally for these events, P was set to 0 on day $t + 1$ to account for culling and movement restriction. Simulations were also carried out using separable w_{index} and w_{test} periods for index cases (2003) and testing (2004-5) premises respectively, to allow for different infectious and exposed periods. Compartmentalising w in such a way did not affect the model results. Where $\mathcal{Y}$ is the set of all premises not assigned as index cases, on any day in 2004, model likelihood was calculated as

$$L = \prod_{i \in \mathcal{Y}} P_i^{Y_i} \left(1 - P_i\right)^{(1 - Y_i)}.$$

A similar approach was used by Keeling et al. (2001). Likelihoods are not directly comparable between models with different w, since this affects not only the

modelled probabilities P, but also the data Y.

A5. Parameter fitting

Maximum likelihood parameter estimates for μ, β, γ, and r were obtained, with confidence limits provided by a Markov-chain Monte Carlo algorithm. An empirical adjustment to P was made before calculating L to account for rounding errors leading to unit probabilities for a small number of premises, which would lead to undesirable zero likelihood statistics.

$$P'_i = \frac{P_i + \delta}{1 + 2\delta}$$

where $\delta = 10^{-10}$. Goodness of fit of the model was then expressed in terms of log-likelihood, and different model frameworks contrasted using the Akaike Information Criterion (AIC) (Akaike, 1974):

$$AIC = 2k - 2\ln L,$$

where k is the number of parameters fitted. Models without background-rate spread can be considered nested within those with background-rate spread, and models with low cattle-to-cattle transmission nested within those with high cattle-to-cattle transmission. In these cases, AIC selection is equivalent to significant differences in a likelihood ratio test. Elsewhere, the various model frameworks are not nested.

A two-stage process of parameter fitting by minimising negative log-likelihood was performed. First, the Downhill Simplex 'Amoeba' algorithm (Nelder & Mead, 1965) was used to find an approximate solution. The model likelihood surface was found to be rough at small scale across the r axis, therefore an adaptive Metropolis-Hastings algorithm (MH; Hastings, 1970) was used to explore this region of parameter space further. In practice, this did not alter the conclusions drawn by the 'Amoeba'-derived parameter estimates.

Both algorithms were used to fit model parameters β, μ, γ, and where applicable, r. Since the algorithms are inherently unbounded, boundaries were imposed on the model parameters using a logistic function:

$$x' = \frac{x_{\max}}{1 + e^{-x}} \qquad x \in (-\infty, \infty)\,; \qquad x' \in (0, x_{\max})$$

where parameter x is fitted but mapped into parameter x' for use in the model, with range $(0, x_{\max})$. For the 'Amoeba' algorithm, occasional restarts were necessary to avoid local minima.

The Metropolis-Hastings algorithm was used to explore parameter space around the best-fit parameters through Markov-chain Monte Carlo simulation (MCMC). A point in parameter space $\mathbf{x}$ is established where the maximum in model log-likelihood is found $\ln L_{\mathbf{x}}$. A new proposed point is selected by adding a scaled random deviate z chosen from the standard normal distribution to each parameter: $\mathbf{x}'_i = \mathbf{x}_i + z(\mathbf{s}_i + s_{\min})$. The deviate z is scaled according to $\mathbf{s}_i$ with a small number added to prevent the algorithm from initially becoming 'stuck'. The difference d in log-likelihood is calculated: $d = \ln L_{\mathbf{x}'} - \ln L_{\mathbf{x}}$. If $d < 0$ or $u < e^{-d}$ (where u is a number chosen uniformly in the range $[0, 1]$), then $\mathbf{x}'$ is accepted as

the new point, otherwise x is retained. Of all accepted points in the chain so far, the mean $\bar{x}_i$ and variance s_i^2 are calculated. This variance is used to choose the next proposed point as shown above.

Profile confidence intervals were obtained from the set of visited points. A point x lies within the 95 % confidence interval of the parameter estimates where $\ln L_{\mathbf{x}} - \ln L_{\max} < -\chi^2_{\text{crit}}$ where $\chi^2_{\text{crit}} = \frac{1}{2}\chi^2_{0.05[n]}$ where n parameters are fitted.

Preliminary analysis showed that parameter confidence intervals obtained by MCMC were insensitive to the day chosen for evaluating L. Therefore, the average $\ln L$ across all days within 2004 was also used to get an overall indication of goodness of fit (Table 1, Fig. 1, main text). It is not possible to treat Y_i between days as independent as it is strongly serially correlated.

The low-within-herd-transmission model required re-extraction and batching of the movement data for each definition of an high-risk area used. Therefore, the radius parameter r could not be automatically fitted by Amoeba or MCMC, and a limited selection of r values was explored between 3150 and 8000 m, concentrated between 4400 and 5400 m. Similarly, full confidence intervals for r cannot be obtained under the low-within-herd transmission model. Those shown in Table 2 (main text) were obtained using MCMC at a fixed radius r of 6000. Other radii examined produced fits with significantly lower likelihoods and they did not contribute to the confidence region.

A6. Modelling over longer timescales

With many premises only being tested once every four years, it is necessary to test the effect of the implicit assumption in the model runs above that no breakdowns can be attributed to movements that occurred before 2002. This therefore involves modelling from an earlier start date. To extend the model for running over considerably longer timescales, it is necessary to treat the high-risk areas as dynamic, not static. Simulations were carried out running the model from the beginning of 2000, with parochial high-risk areas modelled following the distributions of one- and two-year testing interval parishes through the duration of the model seeding period (2000-2003). These were then left unchanged for the testing period (2004). Index cases were specified as all premises with breakdowns occurring in a (dynamic) high-risk area, throughout the seeding period. The parameter w was also assigned dynamically, to equal the current testing period frequency of each parish.

A7. Model robustness

Model results were insensitive to the model start date and the infectious period length w within the range 70 to 365 days (Fig. A1). It might be expected that M would decrease for small w where the movements data become sufficiently rarified as to become uninformative. However, there is much repeated trading within the movement network and a period of movement data of length 70 days (the smallest w examined), already contains a substantial fraction of the premises, particularly for breakdown premises (Fig. A2).

The model design assumes that the rates of infection through high-risk area transmission and background rate transmission are equal for all premises within and without the high-risk areas. This is unlikely, but there is little data available

to quantify the relative risk of bovine TB across farms more precisely. It is important however to consider the potential impact of relaxing the assumption of homogeneity upon the parameters obtained through model fitting. The most parsimonious assumption is that breakdown farms are in some way more susceptible than the population average. All breakdown premises were considered more susceptible to infection by all three routes according to a multiplication factor between one and eight, as shown in Fig. A3. Parameter values obtained by fitting these models were similar to those obtained from the original model. An extreme model assumption that breakdown farms alone were susceptible failed to produce parameter estimates with any confidence.

An alternative assumption is that larger farms are more susceptible. Examination of the distributions of farm sizes as recorded by the CTS and of breakdowns indicates that breakdown risk can be well approximated as being proportional to the logarithm of the mean number of cattle held on a premises (2003 data; Fig. A4). Therefore, susceptibility weightings were assigned to farms on this basis and the model refitted (Fig. A3). For premises with mean cattle held in excess of one, susceptibility in the model was set to its logarithm, and to zero for all other premises. These susceptibilities were then normalised by dividing each by the mean before running the model. Again, modelling heterogeneity in this manner does not greatly affect the parameter estimates, nor is it associated with a lower AIC value (15620).

A modified Hosmer-Lemeshow (HL) goodness of fit test was performed on this final model (Hosmer & Lemeshow, 1995). Premises were ranked according to probability of infection P on the final day of the model run, and partitioned into $k = 10$ groups of similar P such that $\sum P$ was equal across the k groups. Observed $O = \sum Q$ and expected $E = \sum P$ numbers of breakdown premises and the total number of premises n were identified for each group i. The HL test statistic was then calculated as

$$G_{HL}^2 = \sum_{i=1}^{k} \left(\frac{(O_i - E_i)^2}{E_i \left(1 - \frac{E_i}{n_i}\right)} \right)$$

which is compared to a χ^2 distribution with $k - 2$ degrees of freedom. A test statistic of $G_{HL}^2 = 304$ was sufficient to indicate lack of fit within the model, but with c. 130 000 premises, the sensitivity of the test was extremely high. More informative are the values of O/E for each group, which identifies some underestimation of breakdown risk at both the high and low extremes of P. This may suggest further risk factors for BTB not yet identified.

A8. References

Akaike, H. 1974. A new look at the statistical model identification. IEEE Trans. Automatic Control **19**, 716 – 723.

Gilbert, M., Mitchell, A, Bourn, D., Mawdsley, J., Clifton-Hadley, R. et al. 2005. Cattle movements and bovine tuberculosis in Great Britain. Nature **435**, 491 – 496.

Green, D.M., Kiss, I.Z., & Kao, R.R. 2006. Modelling the initial spread of foot-and-mouth disease through animal movements. Proc. R. Soc. Lond. B **273**, 2729 – 2735 doi:10.1098/rspb.2006.3648.

Hastings, W.K. 1970. Monte Carlo sampling methods using Markov chains

and their applications. *Biometrika* **57,** 97 – 109.

Hosmer, D.W., & Lemeshow, S. 1995. Applied Logistic Regression, 2nd Edition. New York, John Wiley & Sons.

Kao, R.R., Danon, L., Green, D.M, & Kiss, I.Z. 2006. Demographic structure and pathogen dynamics on the network of livestock movements in Great Britain. Proc. R. Soc. Lond. B **273**, 1999 – 2007.

Keeling, M.J., Woolhouse, M.E.J., Shaw, D.J., Matthews, L., Chase-Topping, M., Haydon, D.T., Cornell, S.J., Kappey, J., Wilesmith, J. & Grenfell, B.T. 2001. Dynamics of the 2001 UK foot-and-mouth epidemic: Stochastic dispersal in a heterogeneous landscape. *Science* **294,** 813 – 817.

Nelder, J.A. & Mead, R. 1965. A simplex method for function minimization. *Comp. J.* **7,** 308 – 313.

Author contributions

This project was conceived by RRK. The analysis was designed by DMG and RRK, and implemented by DMG. IZK advised on the model analysis. APM supplied and interpreted Vetnet data. The manuscript was written by DMG and RRK. All authors discussed the interpretation of results and commented on the manuscript.

Tables and Figures

Table A1. Comparison of model results for 2004 and 2005. Proportional contributions of the three modelled routes of infection, and the high-risk area radius r (low within-herd spread model.

start year	test year	movements	background	high-risk	radius r, m
2002	2004 [a]	16 %	9 %	75 %	6000
2003	2005 [a]	13 %	9 %	78 %	6000
2000	2004 [b]	18 %	23 %	59 %	–

[a] Low within-herd spread model. [b] Model with dynamically generated high-risk areas.

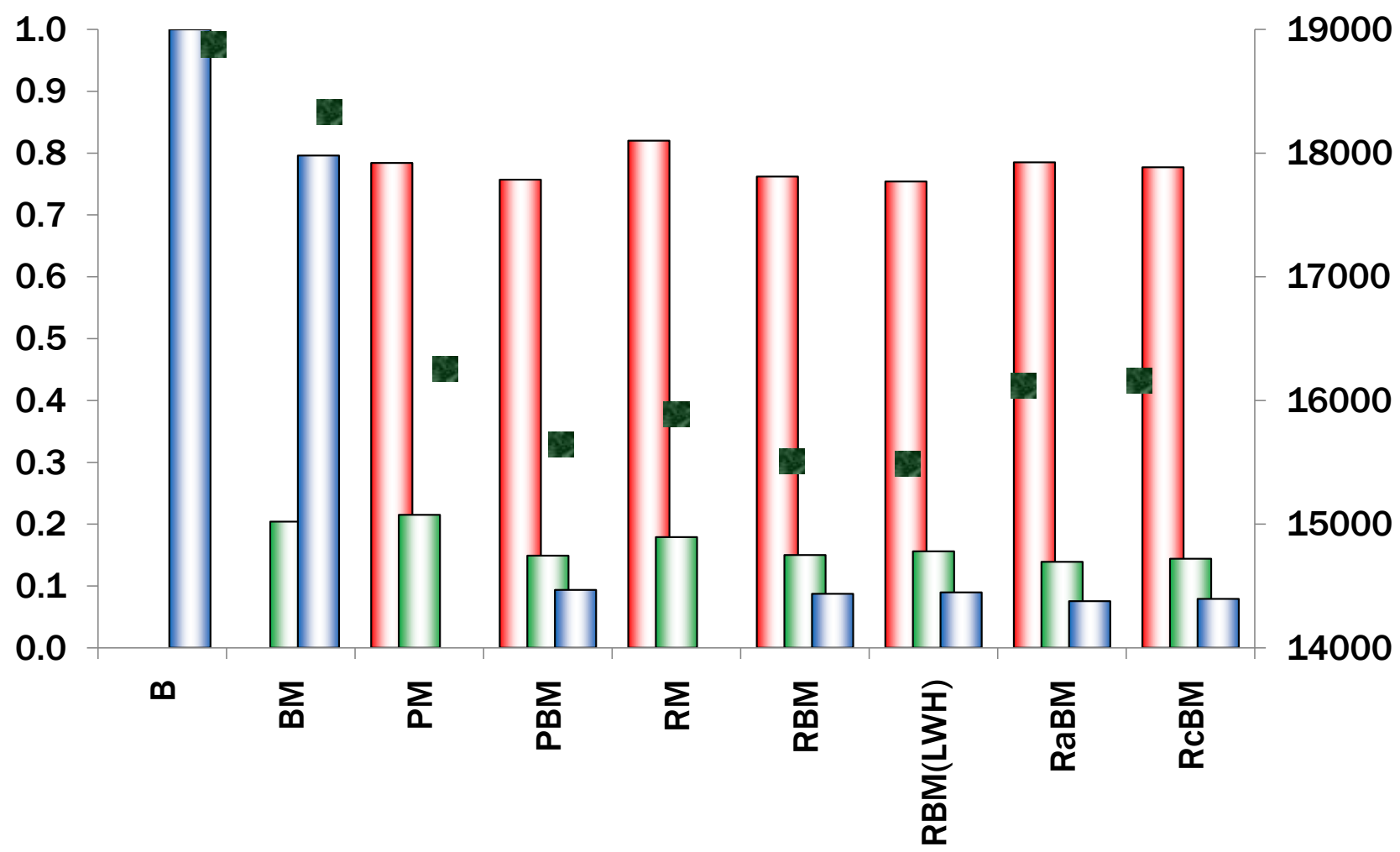

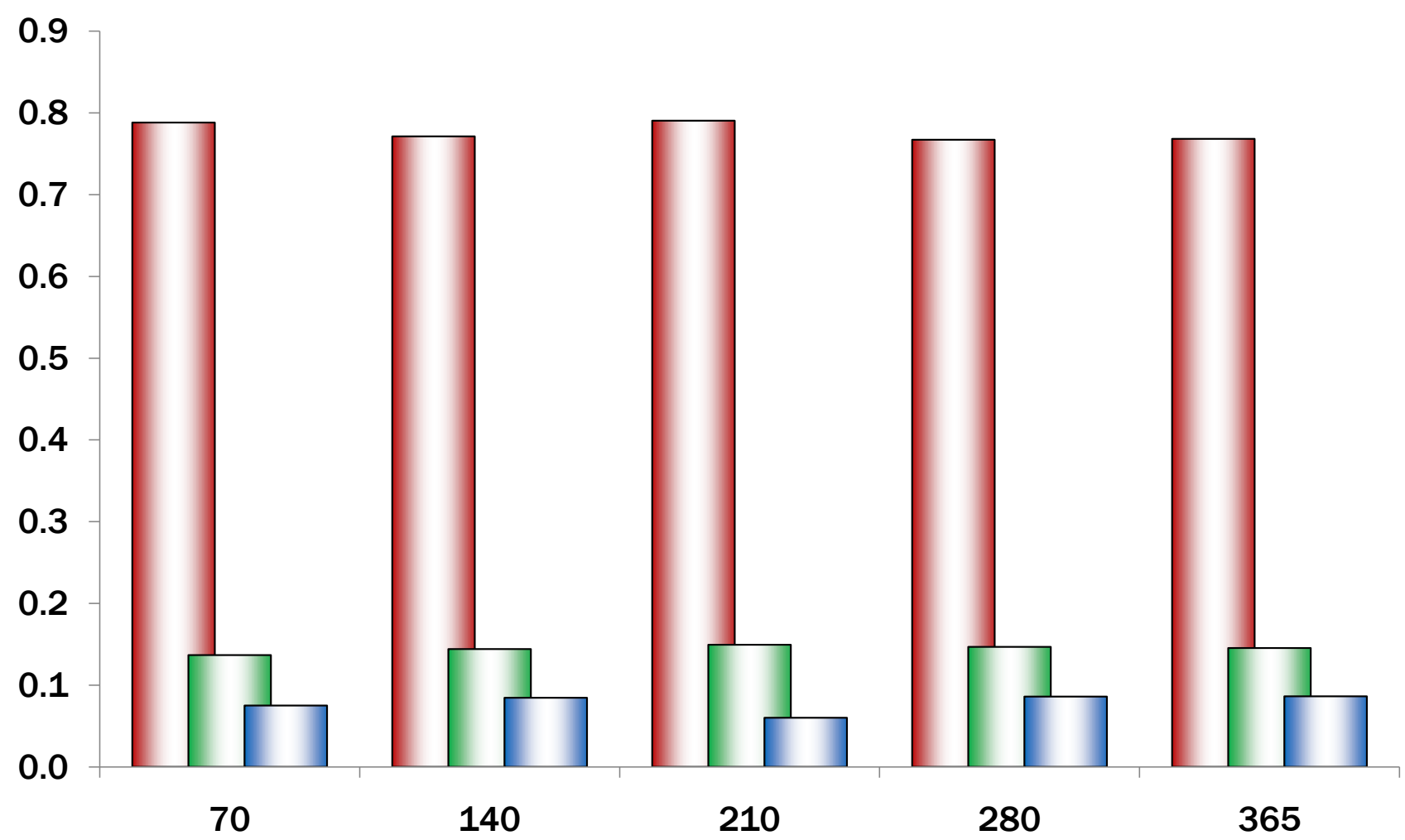

Figure A1. Proportional contributions of high-risk area (red, left series), movement (green, middle series) and background (blue, right series) spread. Best fit models for different model constructions (above) with model AIC values (right-hand axis, symbols); best fit models for five different w (infection window) values (below).

B Background-rate spread fitted; P Parish-based high-risk areas fitted; R Radius-based high-risk areas fitted; M Movement transmission fitted. (a) based on the 'true' index cases; (c) based on the respective randomised index cases. LWH Low within-herd spread model fitted (as Table 1 in main text).

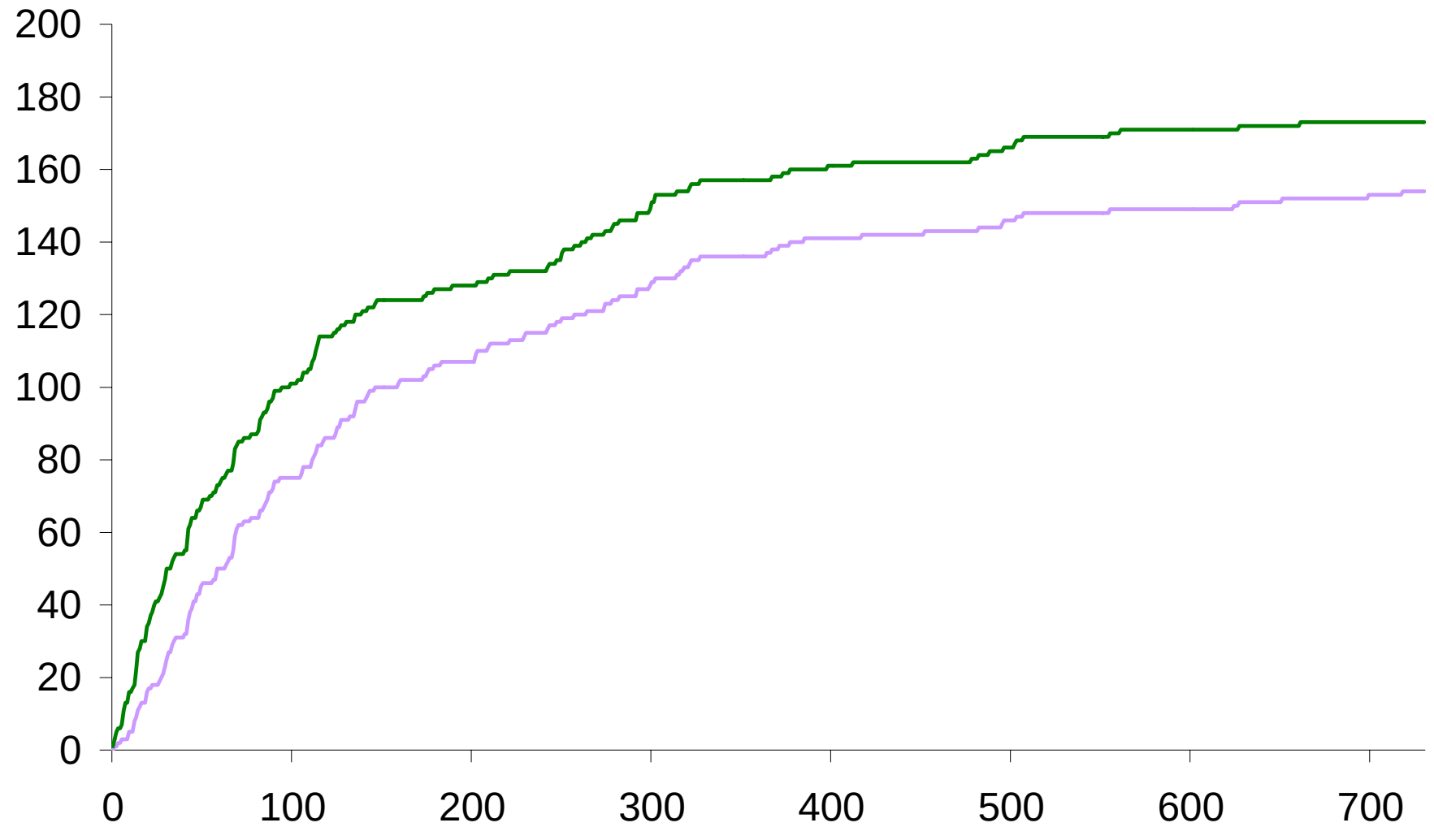

Figure A2. Growth over time of movement network. Numbers of 2004 breakdown premises outside of best-fit high-risk areas present in x days (x-axis) of movement records beginning Jan. 2003. Green (upper line): premises encountered as destinations of any movements; lilac (lower line): premises encountered as destinations of cattle previously moved through high-risk areas (as used by low within-herd transmission model). The numbers of herds encountered by "high risk" movements saturates, suggesting that inclusion of additional days of movement data is unlikely to increase the proportion of breakdowns explained by movements.

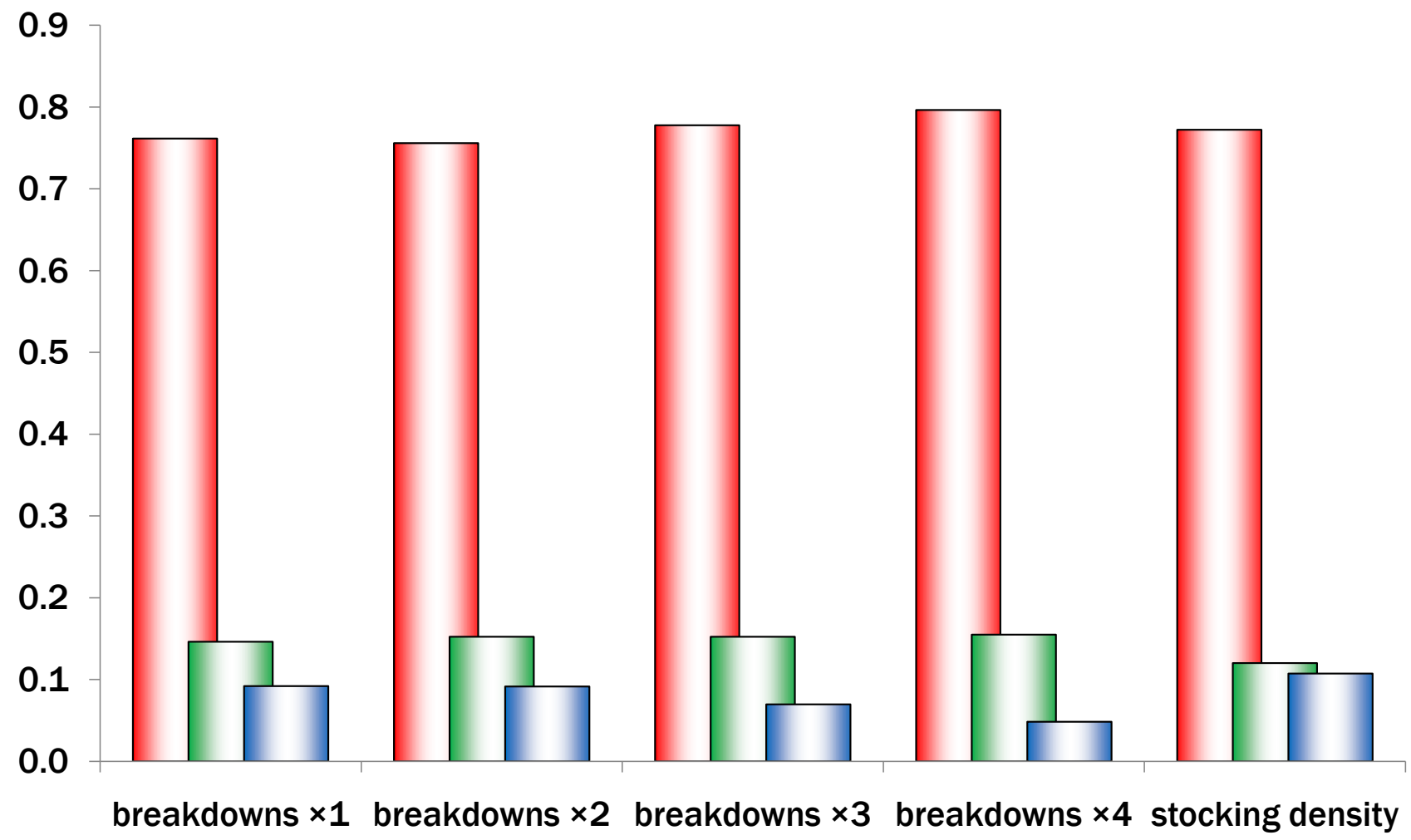

Figure A3. Sensitivity analysis. Estimates for proportions of infections caused by high-risk area (red, left series), movement (green, middle series), and background (blue, right series) transmission. Column blocks one to four: All breakdown cases were assumed proportionally more susceptible by the proportion indicated. Column block five: 2003 stocking density used to estimate relative susceptibility.

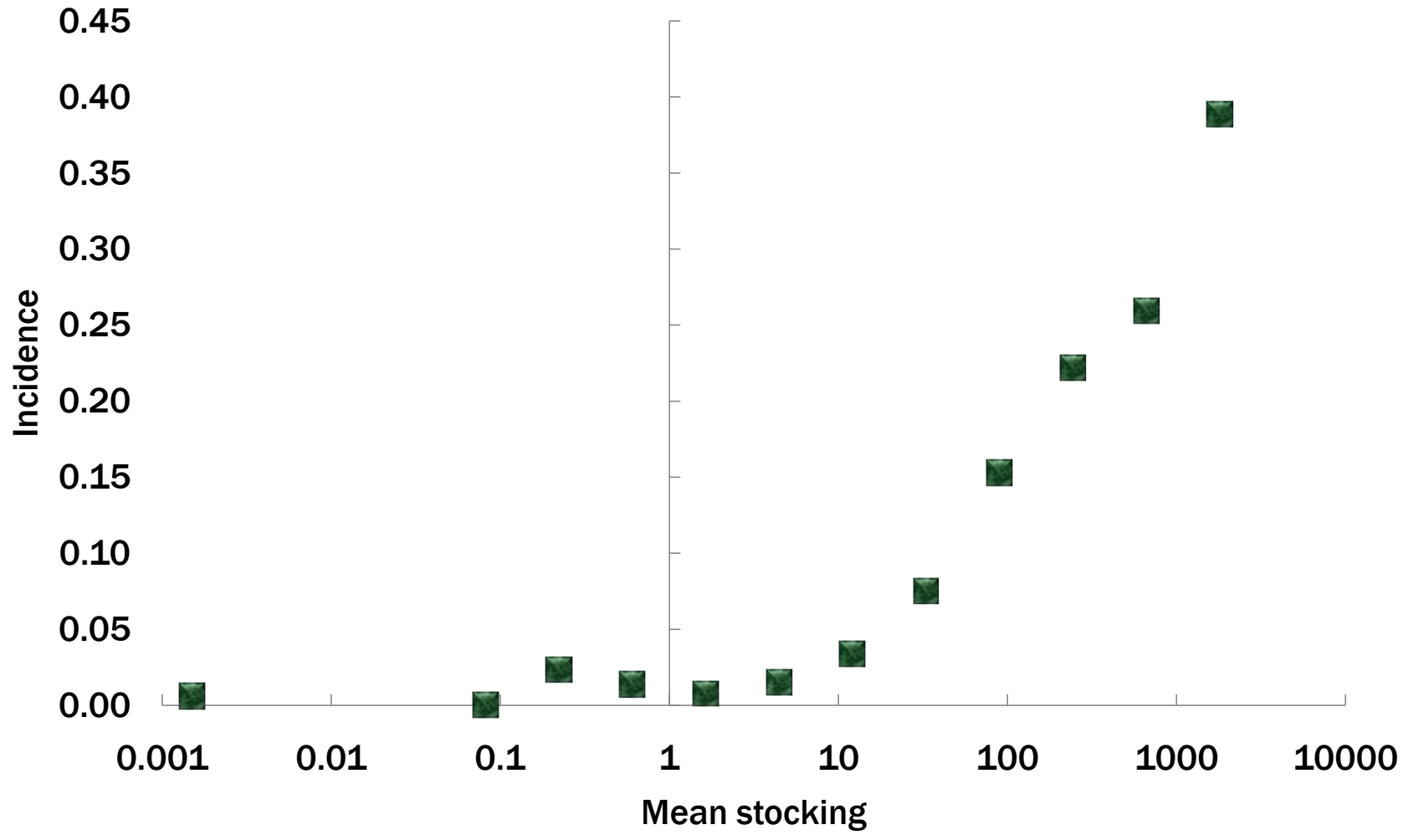

Figure A4. Incidence as a function of farm size. Histogram showing relative incidence of BTB versus binned total animal years per premises (2003 movement data). There is an approximately log-linear relationship above one animal year.

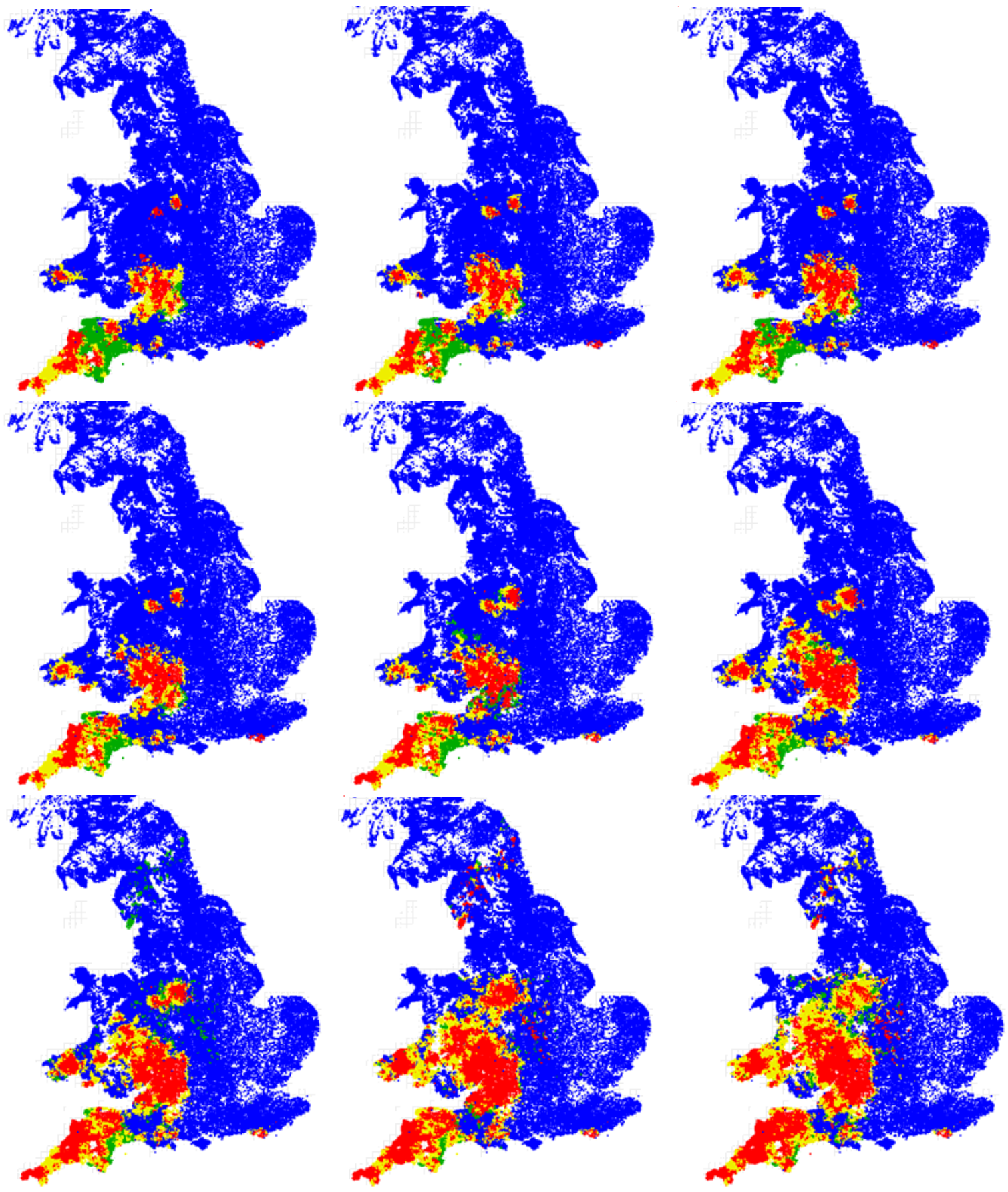

Figure A5. Distribution of parochial testing frequencies from 1998 to 2006. Top row: 1998-2000; middle: 2001-3; bottom: 2004-6. 1-, 2-, 3- and 4-year testing frequencies are indicated by red, yellow, green, and blue respectively (white: no farms).

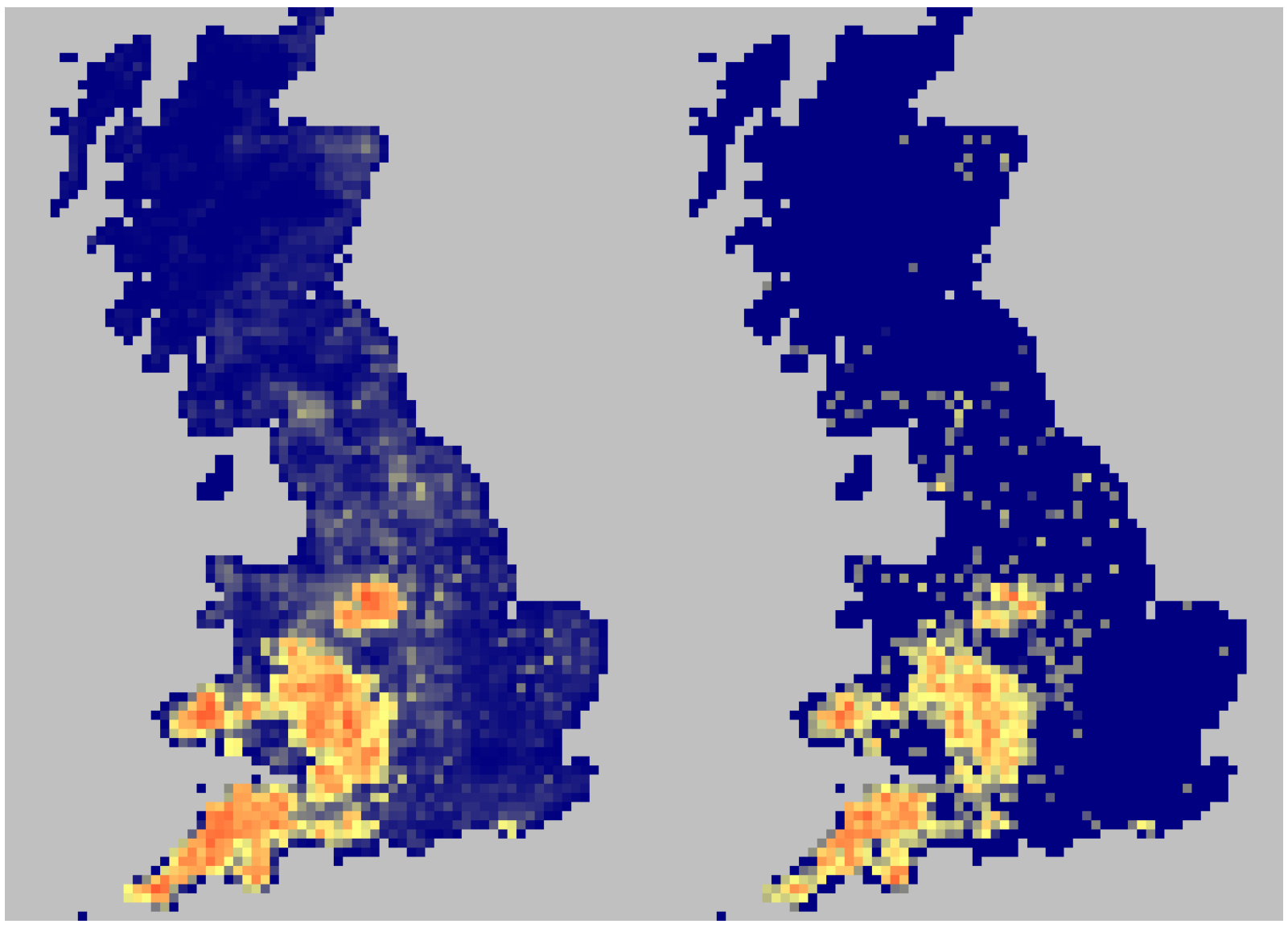

Figure A6. Observed and predicted BTB distribution. Density of 2004 infections (blue, low; red, high) in 100 km^2 squares from best-fit model with low within-herd infection. Left panel: estimated BTB state P derived from model output; right panel: assumed BTB state Y derived from data.
